# Functional characterization of a constitutively active kinase variant of *Arabidopsis* phototropin 1

**DOI:** 10.1074/jbc.M117.799643

**Published:** 2017-06-29

**Authors:** Jan Petersen, Shin-ichiro Inoue, Sharon M. Kelly, Stuart Sullivan, Toshinori Kinoshita, John M. Christie

**Affiliations:** From the ‡ Institute of Molecular, Cell, and Systems Biology, College of Medical, Veterinary, and Life Sciences, University of Glasgow, Bower Building, Glasgow G12 8QQ, United Kingdom,; the § Division of Biological Science, Graduate School of Science and; ¶ Institute of Transformative Bio-Molecules (WPI-ITbM), Nagoya University, Chikusa, Nagoya 464-8602, Japan

**Keywords:** Arabidopsis thaliana, ATP, autophosphorylation, flavin mononucleotide (FMN), serine/threonine protein kinase, A'α-helix, LOV domain, phototropin

## Abstract

Phototropins (phots) are plasma membrane–associated serine/threonine kinases that coordinate a range of processes linked to optimizing photosynthetic efficiency in plants. These photoreceptors contain two light-, oxygen-, or voltage-sensing (LOV) domains within their N terminus, with each binding one molecule of flavin mononucleotide as a UV/blue light–absorbing chromophore. Although phots contain two LOV domains, light-induced activation of the C-terminal kinase domain and subsequent receptor autophosphorylation is controlled primarily by the A′α-LOV2-Jα photosensory module. Mutations that disrupt interactions between the LOV2 core and its flanking helical segments can uncouple this mode of light regulation. However, the impact of these mutations on phot function in *Arabidopsis* has not been explored. Here we report that histidine substitution of Arg-472 located within the A′α-helix of *Arabidopsis* phot1 constitutively activates phot1 kinase activity *in vitro* without affecting LOV2 photochemistry. Expression analysis of phot1 R472H in the phot-deficient mutant confirmed that it is autophosphorylated in darkness *in vivo* but unable to initiate phot1 signaling in the absence of light. Instead, we found that phot1 R472H is poorly functional under low-light conditions but can restore phototropism, chloroplast accumulation, stomatal opening, and leaf positioning and expansion at higher light intensities. Our findings suggest that *Arabidopsis* can adapt to the elevated phosphorylation status of the phot1 R472H mutant in part by reducing its stability, whereas the activity of the mutant under high-light conditions can be attributed to additional increases in LOV2-mediated photoreceptor autophosphorylation.

## Introduction

Plant growth and development is intricately coordinated by a range of photoreceptor systems that respond to light quality, duration, and intensity. These include the red/far-red light–sensing phytochromes (phyA–E), the UV-B–specific photoreceptor UVR8, and several flavin-based blue light receptors (1). The latter is comprised of three different photosensory proteins: the cryptrochromes, the Zeitlupe family, and the phototropins (2). *Arabidopsis* contains two phototropins (phot1 and phot2) that control a variety of processes involved in optimizing photosynthetic light capture. These include phototropism, leaf positioning and expansion, chloroplast relocation movement, and stomatal opening (3). Consequently, phot-deficient mutants of *Arabidopsis* are compromised in their biomass under low-light conditions because of reduced photosynthetic productivity (4).

Phots^2^ are plasma membrane–associated serine/threonine kinases that exhibit receptor autophosphorylation following blue light irradiation (5). Although autophosphorylation occurs on multiple sites throughout the protein (6, 7), phosphorylation of two serine residues within the activation loop of the kinase domain has been reported to be essential for phot function (6, 8). Autophosphorylation of phots can be monitored *in vitro* in the presence of radiolabeled ATP following their heterologous expression in insect cells (9–11). Light regulation of phot kinase activity is mediated by a photosensory region within the N terminus of the protein. This region contains two light-, oxygen-, or voltage-sensing (LOV) domains that bind oxidized flavin mononucleotide (FMN) as a UV/blue light–absorbing cofactor (12). The FMN chromophore is bound non-covalently within the center of the LOV domain formed by several α-helices and a five-stranded β-sheet scaffold, a structure that is characteristic for members of the Per-ARNT-Sim (PAS) superfamily (13). Upon photoexcitation, a flavin triplet state is produced (14) that subsequently leads to the formation of a covalent bond between the FMN isoalloxazine ring and a conserved cysteine residue (15, 16). Formation of this flavin–cysteinyl adduct is a hallmark of LOV domain photochemistry and is fundamental for triggering the activation of this protein-based photoswitch (17). Although phots contain two LOV domains, LOV2 functions as the principal light sensor regulating receptor autophosphorylation and signaling (18–20). In contrast, LOV1 appears to modulate the action of LOV2 (19, 21, 22) and may also be involved in mediating receptor dimerization (23).

A central role for the LOV2 domain in regulating phot kinase activity is derived from its position within the photoreceptor molecule. Helical segments flanking the N and C terminus of the LOV2 core, known as A′α and Jα, respectively (24), are important in this regard (2, 17). The A′α-LOV2-Jα region is proposed to form a closed or inactive conformation with the C-terminal kinase domain and acts to repress phot kinase activity in darkness (21). Light sensing by LOV2 results in structural changes in A′α (25) and Jα (26), which alleviate this repressive and promote ATP binding to the kinase domain (27). Side chain rotation of a conserved glutamine residue within the LOV domain has a central role in propagating these structural alterations at the β-sheet surface (28). FMN–cysteinyl adduct formation results in the flipping of this side chain to temporarily alter its hydrogen bonding with the FMN chromophore (29). As a result, mutation of this glutamine within the LOV2 domain attenuates light-activated disordering of the Jα-helix (30, 31) and consequently impacts light-induced autophosphorylation of *Arabidopsis* phot1 *in vitro* (31, 32).

Disrupting interactions between the A′α or Jα and the LOV2 core through site-directed mutagenesis has been shown to uncouple its photoregulatory mode of action. The Jα-helix is amphipathic in character, consisting of a polar and hydrophobic side, the latter of which docks onto the β-sheet surface of the LOV2 core (24, 26). Mutation of Ile-608 to glutamate at the apolar side of Jα in *Arabidopsis* phot1 results in receptor autophosphorylation in the absence of light (20, 32, 33). Similarly, mutations in A′α diminish the ability of LOV2 to repress the kinase activity of *Chlamydomonas* phot (34). A′α is amphipathic like Jα and binds to the LOV2 core via hydrophobic interactions in darkness (24). Molecular dynamic simulations suggest that A′α interacts with Jα under these conditions, and these interactions are disrupted upon irradiation (25, 35). Time-resolved transient gating experiments, however, indicate that the unfolding of these helices occurs independently (36).

To date, the impact of A′α and Jα mutations on phot1 function in *Arabidopsis* and whether a constitutively active kinase variant can initiate phot signaling in the absence of a light stimulus has not been explored. An arginine-to-histidine substitution in the A′α of tomato phot1 (R495H) is reported to impair photoreceptor signaling (37), but an equivalent mutation in *Chlamydomonas* phot (R210H) has been shown to exhibit constitutive kinase activity *in vitro* (34). In this study, we clarify the role of the A′α-helix in regulating *Arabidopsis* phot1 kinase activation by investigating the impact of the R472H substitution on its photochemical, biochemical, and functional properties. Despite exhibiting constitutive kinase activity *in vitro* and *in vivo*, phot1 R472H was unable to initiate receptor signaling in *Arabidopsis* in the absence of a light stimulus. By contrast, light responsiveness was readily observed in phot1 R472H expressing lines under higher-light conditions. Although these findings demonstrate the importance of A′α in regulating phot1 kinase activity, they also highlight potential limitations in employing A′α- and Jα-helix mutations to constitutively activate phot1 signaling *in planta*.

## Results

### Phot1 R472H is constitutively active in vitro

Our previous studies have shown that the Jα mutation I608E activates phot1 autophosphorylation in the absence of light (20, 32, 33). Such mutations can therefore be used to artificially bypass the requirement of LOV2 to induce phot1 kinase activity. Mutations in A′α have also been shown to uncouple light-dependent kinase regulation (34). *Chlamydomonas* phot purified from *Escherichia coli* can phosphorylate an N-terminal fragment of *Arabidopsis* phot1 as a substrate in a light-dependent manner (21). Mutations in A′α, including R210H elevated substrate phosphorylation in darkness relative to the WT protein (34). However, the impact of these mutations on the autophosphorylation activity of *Chlamydomonas* were not so apparent.

**Figure 1.**
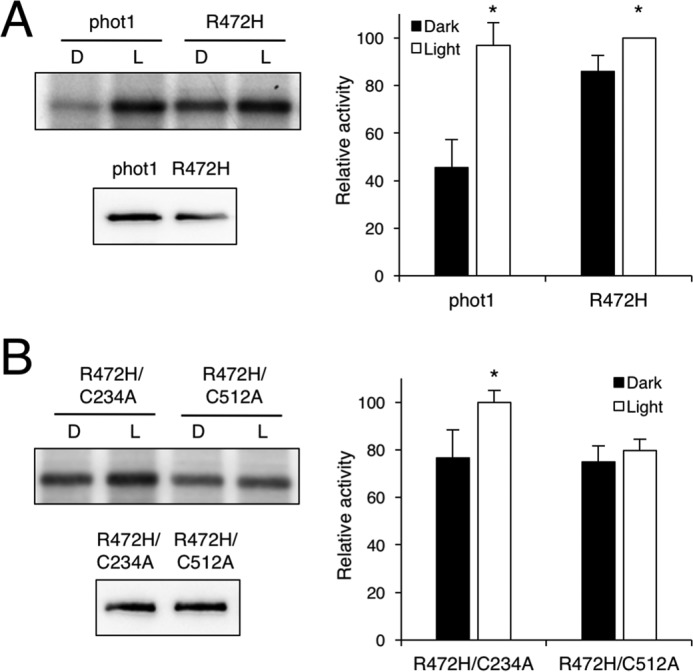
**Effect of the R472H mutation on the autophosphorylation activity of *Arabidopsis* phot1 expressed in insect cells.** *A*, autoradiograph (*left panel*) showing light-dependent autophosphorylation activity of phot1 and the R472H mutant in protein extracts from insect cells. Protein extracts were prepared under dim red light, followed by kinase assays given a mock irradiation (*D*) or irradiated with white light (*L*). Immunoblot analysis of phot1 protein levels is shown below. *B*, impact of photochemically inactivating the LOV domain on phot1 R472H kinase activity. The autoradiograph (*left panel*) shows light-dependent autophosphorylation activity of phot1 R472H harboring the LOV1 mutation C234A or the LOV2 mutation C512A. Immunoblot analysis of phot1 protein levels is shown below. In each case, kinase activity was quantified from the autoradiographs with ImageJ and expressed as a percentage of maximal autophosphorylation (*error bars* indicate ± S.E., *n* = 3). Significant differences between autophosphorylation in the irradiated and mock irradiation control are indicated (*, *p* < 0.05, Student's *t* test).

The autophosphorylation activity of phots can be readily monitored in the presence of radiolabeled ATP following their expression in insect cells (5, 9, 11). We therefore used this approach to examine the impact of the A′α-helix mutation R472H on the kinase activity of *Arabidopsis* phot1. Insect cells infected with a recombinant baculovirus were grown in complete darkness and harvested under dim red light, and total protein extracts were isolated for *in vitro* phosphorylation analysis. As shown in Fig. 1*A*, phot1 exhibits basal levels of autophosphorylation activity in darkness and increases following a brief irradiation with saturating intensities of white light. Replacement of Arg-472 with histidine in the A′α-helix increased receptor autophosphorylation in the absence of light relative to the WT protein, consistent with the proposal that this mutation can uncouple the repressive action of the A′α-LOV2-Jα photoswitch.

### Residual light–induced autophosphorylation in phot1 R472H is mediated by LOV2

Despite exhibiting increased receptor autophosphorylation in darkness, phot1 R472H also showed a remaining level of light-induced autophosphorylation (Fig. 1*A*). Similar residual increases in autophosphorylation have also been observed for *Arabidopsis* phot1 harboring mutations in the Jα-helix (32, 33). To investigate whether the remaining level of light-induced autophosphorylation detected for phot1 R472H could be assigned to LOV1 or LOV2, we incorporated mutations that are known to impair their photochemical reactivity. Incorporation of the C512A mutation in LOV2 was found to abolish light-induced autophosphorylation in phot1 R472H. These findings indicate that the light-induced autophosphorylation observed for phot1 R472H can be attributed to residual LOV2 photoactivity (Fig. 1*B*) (19, 32). Consistent with this conclusion, impairing LOV1 photochemistry by incorporating the C234A mutation did not affect the autophosphorylation activity of the R472H mutant. Together, these findings demonstrate that, although phot1 R472H exhibits autophosphorylation activity in darkness, the A′α-LOV2-Jα photoswitch can still mediate residual responsiveness under saturating light conditions.

### LOV2 R472H exhibits light-induced structural changes

Expression and purification of individual LOV domains in *E. coli* provides a rapid means to monitor the effects of point mutations on their photochemical properties (38). We therefore examined whether the R472H mutation could impact the spectral characteristics of *Arabidopsis* phot1. WT and mutated forms of the LOV1 and 2 region of the phot1 protein, including the A′α- and Jα-helix (amino acids 180–628), were expressed and purified from *E. coli* as N-terminal calmodulin-binding peptide fusions (12). The WT protein and R472H mutant exhibited identical absorption spectra with absorption maxima at 375, 447, and 475 nm (supplemental Fig. S1). In both cases, irradiation resulted in the formation of an FMN–cysteinyl adduct that coincided with a loss of absorption at 447 nm (data not shown). Dark decay of this photoproduct was monitored for both the WT and the R472H mutant. Again, the R472H mutant displayed properties that were indistinguishable from the WT, with dark reversion being completed within 400 s (supplemental Fig. S1). Based on these findings, we conclude that the A′α R472H mutation uncouples the repressive action of LOV2 without impacting its spectral characteristics.

**Figure 2.**
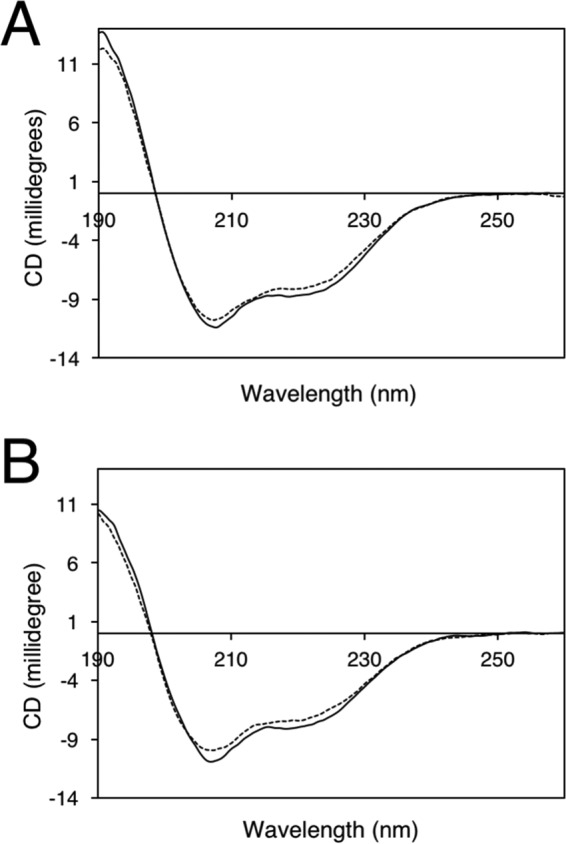
**Light-induced structural changes of phot1 A'α-LOV2-Jα measured by far UV circular dichroism spectroscopy (190–260 nm).** *A*, CD spectra of phot1 A'α-LOV2-Jα in the dark (*solid line*) or after irradiation with blue light (*dashed line*). *B*, CD spectra of phot1 A'α-LOV2-Jα carrying the R472H mutation in the dark (*solid line*) and after blue light treatment (*dashed line*). Data shown for each protein represent the average of two scans, collected at a scan rate of 50 nm min^−1^ using a bandwidth of 1 nm.

CD spectroscopy can be used to probe structural changes associated with the LOV domain in response to irradiation (30, 39). We used this approach to examine the secondary structural content of the A′α-LOV2-Jα region by monitoring its far ultraviolet spectrum. In our hands, the CD spectrum for phot1 A′α-LOV2-Jα exhibited a small but reproducible change in spectral intensity after irradiation with blue light (Fig. 2*A*). These light-induced changes were also apparent for the R472H mutant (Fig. 2*B*). These structural changes are smaller compared with those reported previously for the LOV2-Jα region of oat phot1 (30, 40, 41). However, their occurrence in the R472H mutant correlates well with our insect cell expression data showing that the R472H mutant is still capable of mediating residual increases in receptor autophosphorylation under saturating light conditions (Fig. 1).

### Phot1 R472H is autophosphorylated in darkness in vivo

**Figure 3.**
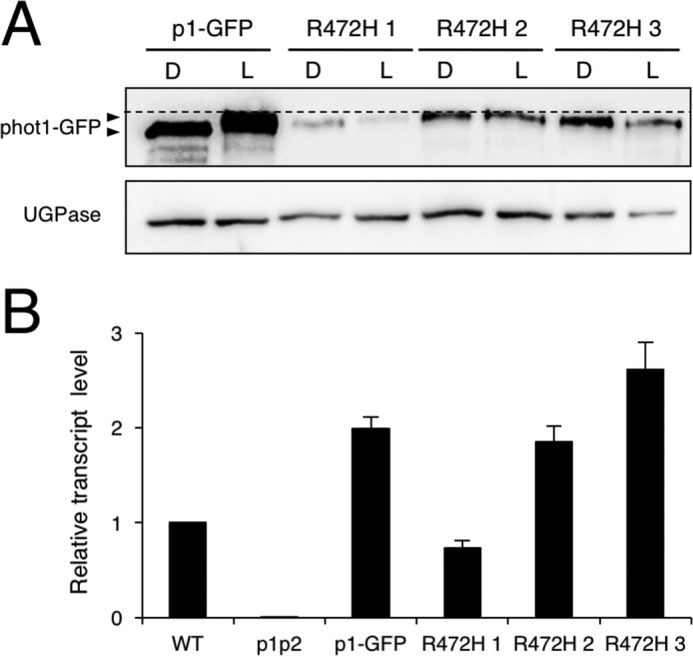
**Expression of p1-GFP R472H in the *phot1 phot2* double mutant (*p1p2*).** *A*, immunoblot analysis of total protein extracts from 3-day-old etiolated seedlings of p1-GFP and three independent p1-GFP R472H transgenic lines (*R472H 1–3*). Total protein extracts were isolated from seedlings either maintained in darkness (*D*) or irradiated with 20 μmol m^−2^ s^−1^ of blue light for 15 min (*L*) and probed with anti-phot1 antibody (*top panel*) or antibody raised against UDP-glucose pyrophosphorylase (UGPase) as a loading control (*bottom panel*). The *dashed line* indicates the highest mobility edge. *B*, quantitative RT-PCR analysis of *PHOT1* transcript levels in each of the different genotypes.

To examine the impact of the R472H mutation *in viv*o, we generated transgenic *Arabidopsis* lines expressing phot1 R472H fused to green fluorescent protein (p1-GFP R472H) in the *phot1 phot2* double mutant under the control of the native *PHOT1* promoter. Three independent p1-GFP R472H lines (1–3) were chosen for analysis, and a transgenic line expressing phot1-GFP (p1-GFP) was included for comparison. Immunoblot analysis of total protein extracts from 3-day-old etiolated seedlings showed that phot1 R472H protein levels were lower in comparison with those observed in the p1-GFP line and in WT *Arabidopsis* seedlings (Fig. 3*A*). The low protein levels observed for phot1 R472H were not a consequence of poor expression because *PHOT1* transcripts were readily detectable in each of the transgenic lines (Fig. 3*B*). It is therefore likely that the R472H mutation has an impact on phot1 protein abundance.

Autophosphorylation of phot1 results in a reduced electrophoretic mobility that can be readily detected by immunoblotting (42). We therefore used this property to determine the autophosphorylation status of phot1 R472H *in vivo*. As shown in Fig. 3*A*, protein extracts were harvested from etiolated seedlings either maintained in darkness or irradiated with blue light (20 μmol m^−2^ s^−1^ for 15 min). Immunoblotting showed a reduction in the electrophoretic mobility for phot1 following irradiation, indicative of receptor autophosphorylation (Fig. 3*A*). Moreover, this analysis revealed that the R472H mutant displays a reduced electrophoretic mobility even in darkness (Fig. 3*A*). These findings therefore demonstrate that this variant exhibits constitutive kinase activity when expressed in *Arabidopsis*.

### Phot1 R472H does not impair photoactivation of early signaling events

**Figure 4.**
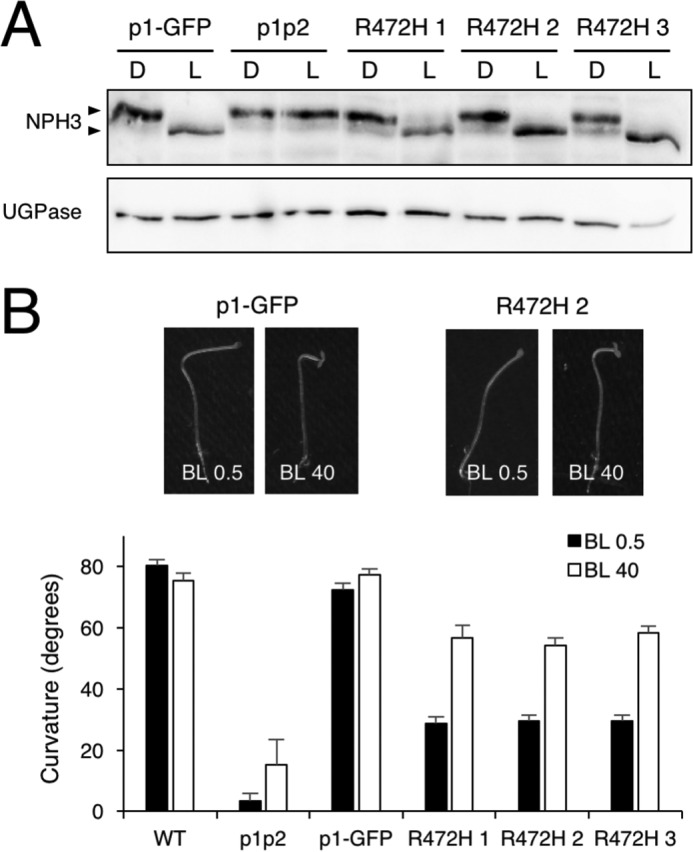
**Impact of phot R472H on phototropic signaling.** *A*, NPH3 phosphorylation status in p1-GFP, *phot1 phot2* (*p1p2*), and p1-GFP R472H transgenic lines. Shown is immunoblot analysis of total protein extracts isolated from 3-day-old etiolated seedlings either maintained in darkness (*D*) or irradiated with 20 μmol m^−2^ s^−1^ of blue light for 15 min (*L*). Protein extracts were probed with anti-NPH3 antibody (*top panel*) or anti-UGPase antibody as a loading control (*bottom panel*). *B*, hypocotyl phototropism in 3-day-old etiolated seedling of the WT, p1p2, p1-GFP, and the p1-GFP R472H transgenic lines. Seedlings were irradiated with unidirectional blue light at 0.5 μmol m^−2^ s^−1^ (*BL 0.5*) or 40 μmol m^−2^ s^−1^ (*BL 40*) for 24 h. Curvatures were calculated as the mean ± S.E. of 40–54 seedlings. Representative images for p1-GFP and R472H 2 are shown.

Because our analysis showed that phot1 R472H is autophosphorylated in darkness in etiolated *Arabidopsis*, we examined whether this activity was sufficient to initiate early events associated with phot1 signaling. Non-phototropic hypocotyl 3 (NPH3) is essential for phototropism in *Arabidopsis* (43–45) and is rapidly dephosphorylated in response to phot1 activation (46). We therefore investigated whether the phosphorylation status of NPH3 is altered in seedlings expressing the R472H mutant. Etiolated seedlings were either maintained in darkness or irradiated with blue light (20 μmol m^−2^ s^−1^ for 15 min), and total protein extracts were harvested for immunoblot analysis. As expected, blue light enhanced the electrophoretic mobility of NPH3 in the p1-GFP line but not in the *phot1 phot2* double mutant (Fig. 4*A*), consistent with it being dephosphorylated in a phot1-dependent manner. Dephosphorylation of NPH3 was also apparent in the p1-GFP R472H lines (Fig. 4*A*). Furthermore, NPH3 was present as its phosphorylated form under dark conditions. These findings demonstrate that phot1 R472H is light-responsive *in vivo* but unable to initiate early signaling events in the absence of light despite already being autophosphorylated.

### Phot1 R472H is functional at higher light intensities

Having demonstrated that phot1 R472H could induce NPH3 dephosphorylation in response to light, we next assessed its ability to restore phototropism in the *phot1 phot2* double mutant. Phot1 can mediate second-positive hypocotyl phototropism over a wide range of fluence rates of directional blue light (10). We first monitored the second-positive phototropism response of etiolated seedlings irradiated with 0.5 μmol m^−2^ s^−1^ of unilateral blue light for 24 h (Fig. 4*B*). For WT and p1-GFP seedlings, hypocotyl curvature reached an angle of ∼80° during this period. However, phototropism was only partially restored in p1-GFP R472H seedlings at these light intensities, reaching a curvature angle of ∼30°. In contrast, the p1-GFP R472H lines displayed a more prominent curvature response (∼60°) when hypocotyls were irradiated with higher intensities of unilateral blue light (40 μmol m^−2^ s^−1^, Fig. 4*B*).

**Figure 5.**
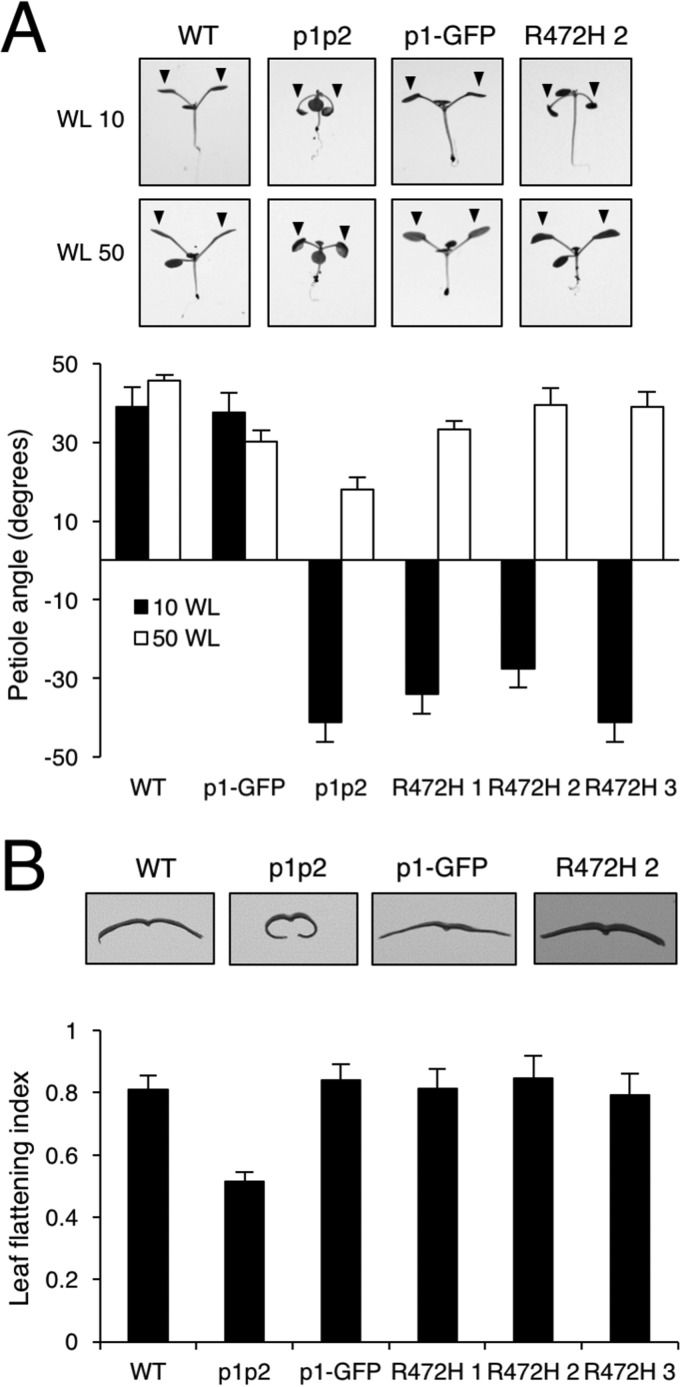
**Phot1 R472H is functional for leaf positioning and leaf expansion.** *A*, leaf positioning in WT, *phot1 phot2* (*p1p2*), p1-GFP, and three independent p1-GFP R472H transgenic lines (*R472H 1–3*). Plants were grown under 80 μmol m^−2^ s^−1^ of white light (16/8 light/dark cycles) for 7 days and then transferred to 10 μmol m^−2^ s^−1^ (*WL 10*) or 50 μmol m^−2^ s^−1^ (*WL 50*) white light (16/8 h light/dark cycle) for 5 days before seedlings were photographed. Petiole angle from the horizontal was measured for the first true leaves. Each value is the mean ± S.E. of 10 seedlings. Representative images of each of the genotype are shown. *B*, leaf expansion responses for each of the genotypes. The leaf expansion index of the fifth rosette leaf was expressed as the ratio of the leaf area before and after artificial uncurling. Each value is the mean ± S.E. of 12 leaves. Images of leaf sections illustrate the leaf expansion phenotype.

In addition to phototropism, phots mediate a variety of responses that combined promote plant growth through maximizing light capture and optimizing photosynthesis (4). These include petiole and leaf positioning, as well as leaf expansion (47). NPH3 is also involved in mediating these responses (47, 48). Petioles of the first true leaves of WT and p1-GFP seedlings grew obliquely upward when irradiated with low-intensity (10 μmol m^−2^ s^−1^) and moderate-intensity (50 μmol m^−2^ s^−1^) white light, whereas the *phot1 phot2* mutant seedlings displayed impaired responses particularly under low-light conditions (Fig. 5*A*). Petiole positioning was not restored in the p1-GFP R472H lines under low white light but detectable at higher light intensities (Fig. 5*A*). Similarly, quantification of the leaf expansion index (the ratio of the leaf area measured before and after manual uncurling of the leaf) showed that all three p1-GFP R472H lines fully complemented the epinastic curled rosette leaf phenotype of the *phot1 phot2* double mutant when grown under higher-light conditions (80 μmol m^−2^ s^−1^, Fig. 5*B*).

**Figure 6.**
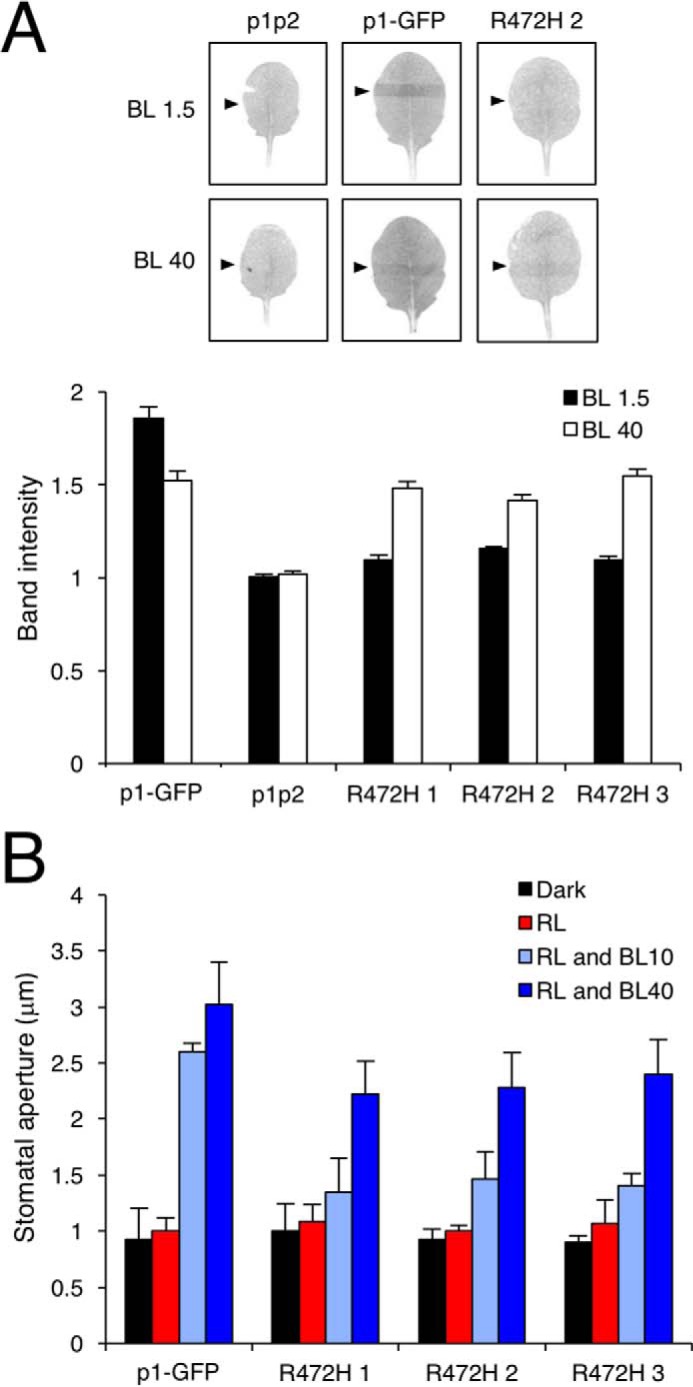
**Phot1 R472H complements chloroplast accumulation movement and stomatal opening at higher light intensities.** *A*, slit band assays of chloroplast accumulation in leaves from *phot1 phot2* (*p1p2*), p1-GFP, and three independent p1-GFP R472H transgenic lines (*R472H 1–3*). Detached rosette leaves were placed on agar plates and irradiated with blue light at 1.5 μmol m^−2^ s^−1^ (*BL 1.5*) or 40 μmol m^−2^ s^−1^ (*BL 40*) through a 1-mm slit for 1 h. *Arrowheads* indicate the irradiated areas. Slit band intensity was quantified using ImageJ, and the relative band intensities were expressed as the ratio of irradiated to non-irradiated areas. Ratios of >1 indicate accumulation. Each value is the mean ± S.E. of 12 leaves. Representative images of each of the genotypes are shown. *B*, blue light–induced stomatal opening responses in p1-GFP plants and each of the p1-GFP R472H transgenic lines. Epidermal strips from dark-adapted plants were irradiated with red light (*RL*, 50 μmol m^−2^ s^−1^) with or without blue light at either 10 or 40 μmol m^−2^ s^−1^ for 3 h. Each value is the mean ± S.E. of 90 stomata, pooled from triplicate experiments.

Chloroplast accumulation movement is mediated by both phot1 and phot2 and contributes to enhancing photosynthetic productivity under low-light conditions (49). The chloroplast accumulation response can be readily monitored by using the slit band assay, where a dark band appears on the leaf when irradiated with low intensities of blue light (1.5 μmol m^−2^ s^−1^) through a 1-mm slit (50). A dark band corresponding to chloroplast accumulation was observed in rosette leaves from WT (supplemental Fig. S2) and p1-GFP plants (Fig. 6*A*). However, p1-GFP R472H lines were not responsive under these light conditions. By contrast, all three p1-GFP R472H lines, in addition to the p1-GFP line, showed chloroplast accumulation at higher fluence rates of blue light (40 μmol m^−2^ s^−1^, Fig. 6*A*), whereas a pale strip was detected in WT seedlings because of phot2-mediated chloroplast avoidance movement (supplemental Fig. S2). Taken together, these physiological studies consistently indicate that the function of phot1 R472H in *Arabidopsis* is more prominent under higher intensities of blue light.

### Phot1 R472H does not promote stomatal opening in darkness or red light

Phots also optimize photosynthesis by regulating stomatal opening in response to blue light (51). Stomatal opening provides another means to assess the functionality of phot1 R472H, especially under conditions other than blue light. We therefore measured the stomatal aperture of epidermal strips in darkness, when irradiated with red light, or when irradiated with red and blue light. The *phot1 phot2* double mutant is deficient in blue light–induced stomatal opening (52). This response was restored in epidermal strips from p1-GFP plants (Fig. 6*B*). No change in stomatal aperture was observed in epidermal strips when plants were maintained in darkness or transferred to red light. Blue light–induced stomatal opening was apparent in the p1-GFP R472H lines but only under higher blue light intensities (Fig. 6*B*). Stomata in p1-GFP R472H lines also did not open in darkness or under red light conditions despite the constitutive kinase activity of the R472H mutant protein.

### Phot1 R472H exhibits intermolecular receptor interactions

**Figure 7.**
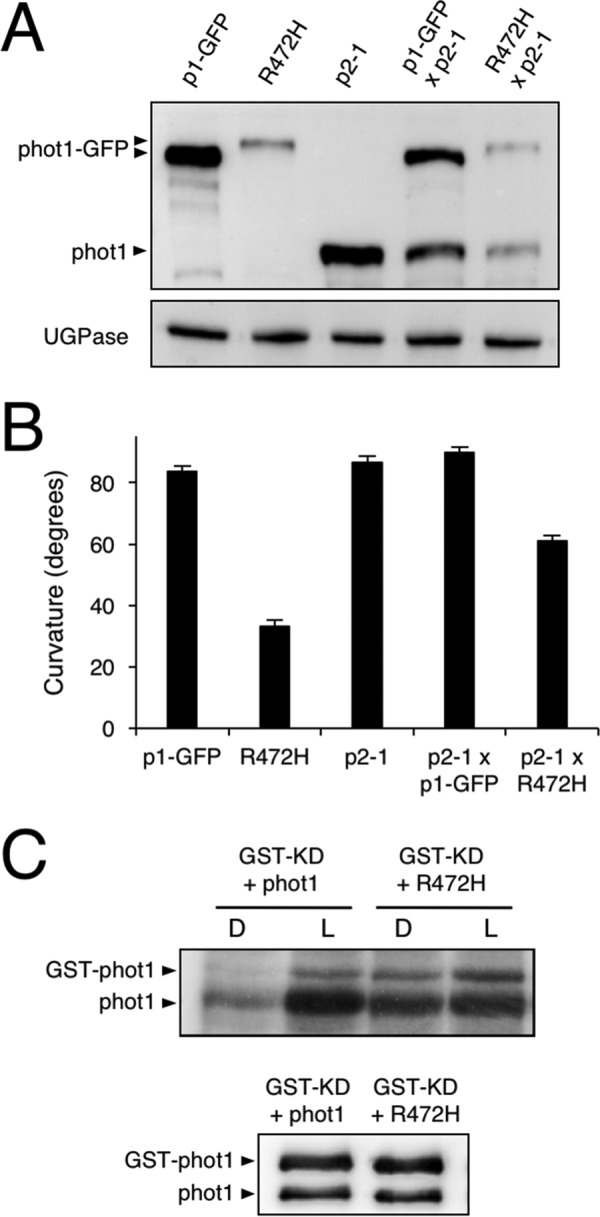
**Impact of phot1 R472H on intermolecular receptor interactions.** *A*, immunoblot analysis of total protein extracts isolated from 3-day-old etiolated seedlings of p1-GFP, p1-GFP R472H (*R472H 2*), *phot2-1* (*p2-1*), and F1 seedlings from p1-GFP × p2-1 and R472H x p2-1 crosses. Protein extracts were probed with anti-phot1 antibody (*top panel*) or anti-UGPase antibody as a loading control (*bottom panel*). *B*, hypocotyl curvature responses of 3-day-old etiolated seedlings for each of the different genotypes. Seedlings were irradiated with unidirectional blue light (*BL*) at 0.5 μmol m^−2^ s^−1^ for 24 h. Curvatures were calculated as the mean ± S.E. of 52–69 seedlings. *C*, transphosphorylation between phot1 R472H and phot1 *in vitro*. The autoradiograph shows transphosphorylation of a kinase-inactive (D806N) version of GST-phot1 (*GST-KD*) by phot1 or phot1 R472H in protein extracts from insect cells. Immunoblot analysis of phot1 protein levels is shown below.

An equivalent A′α mutation (R495H) has been shown to impair the functionality of phot1 in tomato (37). However, these studies were performed in a heterozygous mutant background expressing both WT and R495H versions of phot1. Endogenous phot1 protein levels were severely reduced in this heterozygous background, leading to the proposal that phot1 R495H somehow negatively impacts the stability and function of WT protein. To further investigate this hypothesis, we generated a heterozygous F1 cross between p1-GFP R472H (line 2) and the *phot2-1* mutant. A cross between p1-GFP and *phot2-1* was generated for comparison. We first used these crosses to examine whether the presence of phot1 R472H could affect the abundance of WT phot1 in etiolated seedlings. Immunoblot analysis of total protein extracts from the p1-GFP × *phot2-1* cross showed that protein levels for phot1 and phot1-GFP were comparable (Fig. 7*A*). By comparison, protein levels for WT phot1 were more reduced in the p1-GFP R472H × *phot2-1* cross. We therefore conclude that the presence of phot1 R472H reduces the abundance of WT phot1. We also found that the magnitude of second-positive hypocotyl phototropism was reduced in F1 seedlings of the p1-GFP R472H × *phot2-1* cross, whereas those of the p1-GFP × *phot2-1* cross were unaffected (Fig. 7*B*).

Prolonged irradiation with high-intensity blue light is known to promote phot1 protein turnover (53, 54). Our earlier work has also shown that phot1 autophosphorylation can occur intermolecularly between receptor molecules (20). Continued transphosphorylation of WT phot1 by the R472H mutant could contribute to its reduced protein level in etiolated seedlings (Fig. 6*A*). We therefore used the insect cell expression system to investigate this possibility in more detail. Specifically, we examined whether phot1 R472H could transphosphorylate a kinase-dead (KD) version of phot1 tagged with GST. As found previously (20), WT phot1 is capable of transphosphorylating GST-KD in a light-dependent manner (Fig. 7*C*). Transphosphorylation of GST-KD was also observed in the presence of phot1 R472H, even in the absence of illumination. Hence, these results confirm that phot1 R472H is capable of intermolecular transphosphorylation at least *in vitro*.

## Discussion

### Impact of constitutive kinase activation on phot1 protein stability

Increasing evidence has indicated that light activation of LOV2 results in structural changes in the A'α-helix as well as the to the Jα-helix (36, 55). This work is consistent with such a mechanism, whereby mutations within A'α can disrupt the repressive action of LOV2 on phot kinase activity (34). Moreover, we assessed, for the first time, the functionality of a constitutively active variant of phot1 in *Arabidopsis*.

Our previous attempts to characterize the *in vivo* functionality of a constitutively active kinase variant of phot1 were unsuccessful because of insufficient protein levels despite the presence of detectable transcripts in these transgenic lines (20). Reduced protein levels were apparent in the p1-GFP R472H lines generated here (Fig. 3*A*), making GFP imaging difficult to perform compared with p1-GFP-expressing lines (data not shown). However, *PHOT1* transcripts in the p1-GFP R472H transgenic lines were comparable with those present in WT and p1-GFP seedlings (Fig. 3*B*). These findings, combined with previous work, strongly suggest that constitutive activation of phot1 compromises its stability in *Arabidopsis*. This conclusion agrees with recent results obtained for the non-phototropic seedling 1 (*nps1*) mutant of tomato (37). The *NPS1* locus encodes an R495H variant of phot1, equivalent to the mutant studied here in *Arabidopsis*. This locus severely reduces the protein level of WT phot1 in heterozygous F1 plants (37). Similarly, we found that the presence of phot1 R472H, but not phot1-GFP, could reduce WT phot1 protein levels in the F1 crosses (Fig. 7*A*). These findings provide further support for intermolecular interactions between phot molecules *in vivo*.

Transphosphorylation between phot molecules could account for the results obtained here for the F1 crosses. If constitutive phosphorylation reduces phot1 stability, then transphosphorylation of WT phot1 by R472H would be expected to promote this effect. In support of this hypothesis, we found that transphosphorylation between phot1 R472H and a kinase-inactive version of phot1 can be readily detected *in vitro* (Fig. 7*C*). However, it is worth noting that, although phot1 R472H could reduce the abundance of WT phot1 in the F1 crosses, the electrophoretic mobility of the WT protein did not appear to change (Fig. 7*C*). Further investigation is now warranted to determine sites of intermolecular phosphorylation and their impact on phot1 stability *in vivo*.

### Functionality of phot1 R472H in Arabidopsis

Autophosphorylation within the kinase activation loop of phot1 is essential for signaling (6). Our *in vivo* analysis showed that the R472H mutation was sufficient to promote phot1 autophosphorylation in the absence of light, as evidenced by the reduced electrophoretic mobility in etiolated seedlings (Fig. 3*A*). However, this activity in darkness was not sufficient to promote NPH3 dephosphorylation (Fig. 4*A*). We therefore conclude that, despite exhibiting constitutive kinase activity in darkness both *in vitro* (Fig. 1*A*) and *in vivo* (Fig. 3*A*), phot1 R472H cannot activate receptor signaling in *Arabidopsis*. The R472H mutant was also not capable of promoting stomatal opening in darkness or under red light conditions (Fig. 6*B*). On the contrary, expression of the kinase domain of phot2 in the *phot1 phot2* double mutant was sufficient to open stomata in the absence of a blue light stimulus (56). Activity in darkness was not evident for the p1-GFP R472H lines generated in this study. Instead, phot1 R472H was light-responsive in *Arabidopsis* for multiple responses, albeit under higher-light conditions (Figs. 4–6). The low level of phot1 R472H protein is unlikely to account for its reduced functionality under weak light conditions because transgenic lines expressing phot1 at levels significantly lower than WT have been shown to be fully functional for a range of responses (18, 19, 52, 57). It is therefore possible that the p1-GFP R472H lines have become adapted to the elevated phosphorylation status of R472H so that receptor signaling is not effective under low-light conditions. Functionality for phot1 R472H was observed under higher light intensities for all responses studied here and can be attributed to additional increases in LOV2 photoactivation and receptor autophosphorylation. A residual level of light-induced autophosphorylation was still detectable for the R472H mutant *in vitro* (Figs. 1*A* and 7*C*), and our double mutant analysis attributed this activity to LOV2 and not LOV1 (Fig. 1*B*). Likewise, minor changes in secondary structure were still detectable for the phot1 A′α-LOV2-Jα region harboring the R472H mutation (Fig. 2) in response to irradiation. Taken together, our findings suggest that phot1 R472H does not fully mimic the irradiated state of the receptor protein, as further increases in LOV2 photoactivation and receptor autophosphorylation can occur at higher light intensities.

### The role of A′α and Jα in phot1 activation

Structural changes in both the A′α- and Jα-helix are required for the regulatory action of the LOV2 photoswitch (17). However, it remains unclear how these helical segments are coordinated to regulate phot1 kinase activity. The A'α-LOV2-Jα photoswitch is proposed to function as a as a dark state repressor (2). However, the mechanism of repression and how light-driven conformation changes alleviate this is still not fully understood. Although our studies with *Arabidopsis* phot1 have shown that mutations in either A'α (Fig. 1) or Jα (20, 32, 33) disrupt this repression mechanism, they now demonstrate that such variants do not fully mimic the irradiated state in terms of functionality. Combined mutagenesis of both of these regions may be necessary to completely ameliorate the repressive action of LOV2.

Phosphorylation of two conserved serine residues within the kinase activation loop of phot1 (Ser-849 and Ser-851) are essential for receptor signaling (6). Mutation of these sites to alanine is reported to impair both phot1 (6) and phot2 (8) function in *Arabidopsis*, whereas phosphomimetic substitutions with aspartate are without effect. Interestingly, phot1 mutated in Ser-849 and Ser-851 still displays an electrophoretic mobility shift characteristic of receptor autophosphorylation (6). Thus, we cannot exclude the possibility that these residues are not constitutively phosphorylated in the R472H mutant. If this is the case, this could account for the inability of phot1 R472H to induce receptor signaling in the absence of light. Phosphoproteomic analysis is now required to uncover any differences in the *in vivo* phosphorylation profiles for phot1 mutants in A'α and Jα to fully ascertain how each of these regions contributes to receptor activation and autophosphorylation.

## Experimental procedures

### Insect cell expression

A recombinant baculovirus was generated using the Bacmagic transfection kit (Merck Millipore) in accordance with the instructions of the supplier. Expression of recombinant phot1 was performed as described previously (5, 10). Amino acid substitutions were introduced using the QuikChange site-directed mutagenesis kit (Agilent) with the following primer pairs: phot1 R472H F/R, phot1 C234A F/R, or phot1 C512A F/R (supplemental Table S1).

### In vitro phosphorylation

Kinase assays were performed as described previously (5, 10). Protein extract (10 μg) was either mock-irradiated under red light or treated for 10 s with white light at a total fluence of 30,000 μmol m^−2^. Reactions were performed for 2 min at room temperature and stopped by addition of SDS sample buffer. Trans-phosphorylation assays were performed as described previously (20), except that recombinant proteins were expressed individually in insect cells, and protein extracts (10 μg) were mixed prior to phosphorylation analysis. Data were quantified from three biological replicates.

### Plant material and growth conditions

Wild type (*gl-1*, ecotype Columbia) and *phot1–5 phot2-1* and *phot2-1* mutants have been described previously (58, 59), as have transgenic *Arabidopsis* expressing phot1-GFP (60). Seeds were planted on soil or on Murashige and Skoog salts with 0.8% agar (w/v). After 4 °C treatment for 3 days, seeds were grown in a controlled environment room (Fitotron, Weiss-Gallenkamp) under a 16/8 h 22/18 °C light/dark cycle.

### Spectral analysis

For absorbance spectroscopy, the *Arabidopsis* phot1 LOV1 + 2 (amino acid residues 180–628) was expressed and purified as described previously using the pCAL-n-EK vector (Agilent) to create an N-terminal calmodulin-binding peptide fusion by calmodulin affinity chromatography (20, 31). Absorption spectra were measured using a Shimadzu MultiSpec-1501 diode array spectrophotometer at room temperature. The optical path length was 0.5 cm, and protein concentrations were determined by Bradford protein assay (Bio-Rad) using BSA as standard. For CD measurements, a DNA fragment encoding the A'α-LOV2-Jα region of *Arabidopsis* phot1 (amino acid residues 467–629) was PCR-amplified (supplemental Table S1) and cloned into the pHS vector (61) by Gibson assembly (New England Biolabs) via NcoI and NotI to create an N-terminal tagged His_7_-Strep-SUMO fusion. LOV2 protein was expressed and purified by double affinity chromatography as described previously (61). CD spectra were recorded with a JASCO J-810 spectropolarimeter at a protein concentration of 0.4 mg ml^−1^ using a 0.02-cm path length quartz cuvette. Protein samples were irradiated for 5 s (λ_max_, 443 nm; half-bandwidth, 10 nm; 0.06 W cm^−2^) with a bluephase 16i dental curing light (Ivoclar Vivadent).

### Plasmid construction and Arabidopsis transformation

The plant transformation vector encoding *PHOT1-GFP R472H* under the control of its endogenous promoter was generated by replacing part of the coding sequence of WT *PHOT1* in the binary vector pEZR(K)-LN (60) via SalI and BamHI by Gibson assembly. Constructs were transformed into the *phot1–5 phot2-1* double mutant with *Agrobacterium tumefaciens* as described previously (19). T3 lines containing a single transgene locus were selected for analysis based on segregation of kanamycin resistance.

### Transcript analysis

Total RNA was isolated from 3-day-old etiolated seedlings using the RNeasy Plant Mini Kit (Qiagen), followed by DNase treatment (Turbo DNA-free, Thermo Fisher Scientific) and cDNA synthesis using random hexamers and SuperScript IV reverse transcriptase (Thermo Fisher Scientific). Quantitative RT-PCR was performed using primers for *PHOT1* and the internal reference gene *IRON SULFUR CLUSTER ASSEMBLY PROTEIN 1* (*ISU1*) (supplemental Table S1) with Brilliant III SYBR Green QPCR Master Mix (Agilent) and a StepOnePlus (Thermo Fisher Scientific) real-time PCR system.

### Immunoblot analysis

Proteins were detected by immunoblotting on nitrocellulose membranes with anti-phot1 purified antibodies raised against a peptide at the C terminus of *Arabidopsis* phot1 (18), anti-NPH3 antibody (62), and anti-UDP-glucose pyrophosphorylase (Agrisera AB). Blots were developed with anti-rabbit HRP-conjugated secondary antibody (Promega) and Pierce ECL Plus Western blotting substrate (Thermo Fisher Scientific).

### Phototropism

Three-day-old etiolated seedlings grown vertically on Petri dishes containing Murashige and Skoog agar were exposed to light provided by a fluorescent lamp filtered through one layer of blue Plexiglas for 24 h (20). Images of the seedlings were captured using a scanner and hypocotyl curvature was measured using ImageJ (http://rsb.info.nih.gov/ij/).

### Petiole positioning and leaf expansion

*Arabidopsis* seedlings were grown on soil for 10 days before representative plants were photographed. Measurement of leaf expansion was carried out as described previously (4) from 4-week-old plants grown on soil. Leaf areas were measured before and after uncurling, and the ratio of the curled to uncurled area was designated the leaf expansion index. Leaf area was measured using ImageJ software.

### Chloroplast photorelocation

Measurements of chloroplast positioning were performed as described previously (11). Rosette leaves detached from 3-week-old plants grown on soil were placed on agar plates and irradiated with blue light through a 1-mm slit or placed in darkness for 1 h. The plates were placed on a white light transilluminator and photographed. Band intensities were quantified using ImageJ, and the relative band intensities were expressed as the ratio of irradiated to non-irradiated areas.

### Stomatal opening

Stomatal aperture measurements from the abaxial epidermis were performed as described previously (6) using an BX43 microscope (Olympus).

## Author contributions

J. M. C. and J. P. designed and directed the research. J. P., S. I., and S. M. K. planned and performed the experiments. J. M. C., J. P., S. I. S. M. K., S. S., and T. K. analyzed the data. J. M. C. wrote the manuscript. All authors commented on the manuscript.

## Supplementary Material

Supplemental Data

## Footnotes

2 The abbreviations used are:

phot: phototropin
LOV: light, oxygen, or voltage
FMN: flavin mononucleotide
KD: kinase-dead
UGPase: UDP-glucose pyrophosphorylase
SUMO: small ubiquitin-like modifier peptide.
