# Supplementary material for: Functional characterization of a constitutively active kinase variant of Arabidopsis phototropin 1
Source: J Biol Chem. 2017 Jun 29;292(33):13843–52. doi: 10.1074/jbc.M117.799643 (PMC5566536; doi:10.1074/jbc.M117.799643)
Supplement: Supplemental Data [file supp_292_33_13843__index.html]

Functional Characterization of a Constitutively Active Kinase Variant of Arabidopsis Phototropin 1 — Functional characterization of a constitutively active kinase variant of Arabidopsis phototropin 1 — Impact of A′α on Phot1 signaling — Supplemental Data 

# Functional characterization of a constitutively active kinase variant of *Arabidopsis* phototropin 1

## Supplemental Data

- Supplemental data (.pdf, 119 KB) - Supplemental Figures 1-2 and supplemental Table 1
