# Supplementary material for: Functional characterization of a constitutively active kinase variant of Arabidopsis phototropin 1
Source: J Biol Chem. 2017 Jun 29;292(33):13843–52. doi: 10.1074/jbc.M117.799643 (PMC5566536; doi:10.1074/jbc.M117.799643)
Supplement: Supplemental Data [file 10.1074_M117.799643_jbc.M117.799643-1.pdf]

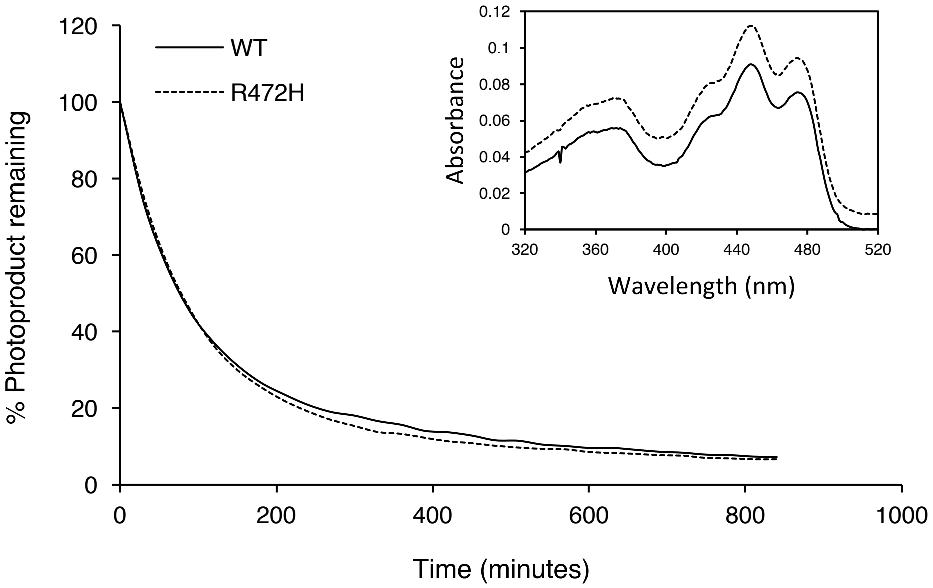

Figure S1. Impact of the R472H mutation on the photochemical properties of phot1 LOV1+2. Dark-recovery kinetics for phot1 LOV1+2 wild-type (WT, solid line) and phot1 LOV1+2 R472H (dashed line). Purified protein was irradiated with a white-light camera strobe flash and the recovery recorded at an absorbance of 450 nm. Recovery is expressed as percentage photoproduct remaining and was calculated from the ratio of absorbance at 450 nm obtained immediately after the light treatment to that obtained for subsequent sampling in darkness. Dark-state absorption spectra for the WT and mutant proteins are shown in the inset.

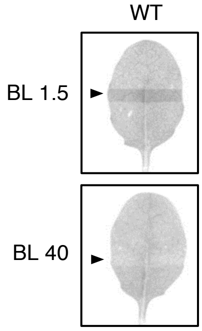

Figure S2. Slit band assays for chloroplast movement responses in leaves from wild-type plants. Detached rosette leaves were placed on agar plates and irradiated with blue light at 1.5 μmol m^{-2} s^{-1} (BL 1.5) or 40 μmol m^{-2} s^{-1} (BL 40) through a 1 mm slit for 1 h. Arrowheads indicate the irradiated areas.

Name	Nucleotide Sequence
phot1 R472H F	CTAGCTACTACACTCGAACATATCGAGAAGAATTTCGTC
phot1 R472H R	GACGAAATTCTTCTCGATATGTTCGAGTGTAGTAGCTAG
phot1 C234A F	CAAAGAAGTCGTCGGCAGAAACGCCCGATTTTTACAAGGAT
phot1 C234A R	ATCCTTGTAAAAATCGGGCGTTTCTGCCGACGACTTCTTTG
phot1 C512A F	TAGCCGTGAAGAAATTCTTGGAAGAAATGCCAGGTTTCTACAAG G
phot1 C512A R	CCTTGTAGAAACCTGGCATTTCTTCCAAGAATTTCTTCACGGCTA
GA pEZ phot1 *Sal*I	AAGATCTAAAAGATGCGTTGTCGACGTTTCAACAAACGTTTGTG
GA pEZ phot1 *Bam*HI	AGCGGCAGCGGCAGCAGCCGGATCCGCAAAAACATTTGTTTGC AGATCTTC
GA pHS A'α-LOV2-Jα WT *Nco*I	AACAGATTGGAGGTGCAACTACACTCGAACGTATCGAG
GA pHS A'α-LOV2-Jα R472H *Nco*I	AACAGATTGGAGGTGCAACTACACTCGAACATATCGAG
GA pHS A'α-LOV2-Jα *Not*I	TCAGTGGTGGTGGTGGTGGTGCTCGAGTGCTTAGTGGTTTGCC CATAAATC
q-PHOT1 F	ATGCCAACATGACACCAGAG
q-PHOT1 R	CCATGGTGGTGAATCTTTCC
q-ISU1 F	GCCATCGCTTCTTCATCTGTTGC
q-ISU1 R	TGGGAGAGAAAGATGCTTTGCG

Table S1. Primers used in this study for mutagenesis, Gibson assembly and qRT-PCR.
